# How do neurons live long and healthy? The mechanism of neuronal genome integrity

**DOI:** 10.3389/fnins.2025.1552790

**Published:** 2025-03-19

**Authors:** Dai Ihara, Nur Rasyiqin Rasli, Yu Katsuyama

**Affiliations:** Division of Neuroanatomy, Department of Anatomy, Shiga University of Medical Science, Otsu, Shiga, Japan

**Keywords:** genome integrity, R-loop, G-quadruplex, DNA repair, topoisomerase

## Abstract

Genome DNA of neurons in the brain is unstable, and mutations caused by inaccurate repair can lead to neurodevelopmental and neurodegenerative disorders. Damage to the neuronal genome is induced both exogenously and endogenously. Rapid cell proliferation of neural stem cells during embryonic brain development can lead to errors in genome duplication. Electrical excitations and drastic changes in gene expression in functional neurons cause risks of damaging genomic DNA. The precise repair of DNA damages caused by events making genomic DNA unstable maintains neuronal functions. The maintenance of the DNA sequence and structure of the genome is known as genomic integrity. Molecular mechanisms that maintain genomic integrity are critical for healthy neuronal function. In this review, we describe recent progress in understanding the genome integrity in functional neurons referring to their disruptions reported in neurological diseases.

## 1 Introduction

The integrity of genomic DNA including DNA sequence and chromatin structure, is essential for cell survival and normal physiological function. Additionally, biochemical modifications such as DNA methylation, histone-associated epigenomic modifications, and other three-dimensional DNA structures play significant roles in maintaining genomic integrity. The genomic integrity must be preserved especially in stem cells, which produce the differentiated cells that constitute each organ, to maintain the physiological functions of each organ and for the health of individuals.

Genomic instability can occur even under normal physiological conditions. DNA damage which can be caused by mutations affect physiological cellular function and systemic health deterioration (Aguilera and García-Muse, 2013). For example, genomic integrity is disrupted in cancer cells, which results in the inability to maintain normal cellular function, as well as uncontrolled proliferation and metastasis. Therefore, the mechanisms that maintain genomic integrity, such as chromatin regulation and DNA repair systems, are crucial for maintaining normal cellular function. A better understanding of these mechanisms, along with strategies for repairing genomic damage, will be essential for disease prevention and treatment (Scheijen and Wilson, 2022).

It has been suggested that genomic integrity is not preserved in mature brain neurons (Zolzaya et al., 2024). 13–41% of human cortical neurons exhibit copy number variants (CNVs) of genes (McConnell et al., 2013), and recent next-generation sequencing studies have revealed that many smaller DNA sequence variants occur in the neuronal genome (Lodato et al., 2018; Luquette et al., 2022). Given that neurons in the brain have an extremely long lifespan and the production of new neurons from neural stem cells is limited, the mechanism preserving the normal function of neurons from genome instability is essential for healthy brain function.

## 2 Instability of the neuronal genome

Neurons must maintain high metabolic activity to transmit information effectively within the nervous system. The brain consumes approximately 25% of the body's glucose to produce the energy needed for this activity (Steiner, 2019; Trigo et al., 2022). Mature neurons produce 4.7 billion molecules of adenosine triphosphate (ATP) per second in their mitochondria. During this process, 1–3% of the oxygen is converted to reactive oxygen species (ROS), which can destabilize the genomic DNA (Salehi et al., 2018). As a result, neurons face higher risks of genome instability than other somatic cells (Zhu et al., 2012; Magistretti and Allaman, 2015). Above all, genomic DNA damage can also occur as a part of normal physiological brain functions. For example, double-strand breaks (DSBs) increase in the entorhinal cortex, parietal cortex, and dentate gyrus during the exploration of a novel environment (Suberbielle et al., 2013). Additionally, DSBs increase in the primary visual cortex when the eyes are illuminated for 15 min (Suberbielle et al., 2013) and in the hippocampus during memory formation in mice (Castro-Pérez et al., 2016).

In addition to physiological activity, neuronal genomics is highly susceptible to damage from drug toxicities (Sanchez-Aceves et al., 2024; Torre et al., 2021; Calls et al., 2021). Alcohol administration has been shown to cause DSBs accumulation in neurons (Rulten et al., 2008). Repeated cocaine administration to mice causes histone hyperacetylation at 1,696 loci in the nucleus accumbens (Renthal et al., 2009), resulting in DNA damage in multiple brain areas (de Souza et al., 2014). Methamphetamine, an indirect adrenergic receptor stimulator, also induces genomic DNA damage in neurons (Johnson et al., 2015; Tokunaga et al., 2008).

Neuronal activity induces the expression of immediate early genes (IEGs) which play important roles in neuronal plasticity (Yap and Greenberg, 2018). Dysregulation of IEGs leads to various neurological disorders (Ebert and Greenberg, 2013). The rapid regulation of IEGs is mediated by physical contact between the enhancer and promoter by single-strand breaks (SSBs) or DSBs in genomic DNA (Madabhushi et al., 2015; Wu et al., 2021; Delint-Ramirez et al., 2022). However, repeated SSBs and DSBs may induce physiological dysfunction of neurons when the 3D structure of the genome is altered (Dileep et al., 2023).

Epigenomic modifications in neurons are required for memory and learning functions in the brain (Zovkic et al., 2013). DNA methylation is modified to cytosines on CpG islands during brain development. The DNA methyltransferase DNMT1 is responsible for the methylation of newly synthesized strands after DNA replication (Li et al., 1992), while DNMT3A regulates gene expression in response to cellular conditions (Wei et al., 2021; Li et al., 2022). DNMT3B, on the other hand, is involved in methylation of X-chromosome-specific genes (Yagi et al., 2020). Proteins that bind to methylated DNA can induce chromatin structural transformation, repressing transcription of downstream genes. Because DNA methylation patterns are disrupted in various malignancies of cancers, DNMT mutations can destabilize the genome (Valencia and Kadoch, 2019). For example, contextual fear conditioning increases DNMT expression in hippocampal neurons, and DNMT inhibition reduces conditioned memory and suppresses long-term potentiation (LTP) in Schaeffer's lateral branch (Levenson et al., 2006). Therefore, DNA methylation regulation plays an important role in neuronal plasticity. Additionally, differences in DNA methylation levels are observed among neurons with different projection sites in the central nervous system (Zhang et al., 2021; Zhou et al., 2023).

The regulatory process of epigenomic modification involves DNA repair with base substitutions by the ten-eleven translocation (TET) enzyme family, TET1, TET2, and TET3. TET enzyme is important to activate the demethylation of DNA involving the process of 5-methylcytosine oxidation to 5-hydroxymethylcytosine (Zhang et al., 2023). It has been shown that spatial learning and short-term memory are impaired in TET1 knockout mice (Zhang et al., 2013), suggesting that maintenance of genomic integrity by regulation of epigenomic modifications is essential for normal neuronal function.

In most organs, malfunctioning cells are removed through apoptosis or phagocytosis, with the replacement of lost cells by regenerative mechanisms. However, in the brain, postnatal neurogenesis is limited only to a few regions, such as the dentate gyrus of the hippocampal formation. Microglia can phagocytose degenerated neurons (Butler et al., 2021), however, the mechanism of removal of neurons as a response to mutations in their genome remains unclear. Genomic mutations in many cell types often result in aberrant cellular characteristics, typically either undergoing apoptosis in a p53-dependent manner or being eliminated by the immune system (Attardi, 2005; Szybińska and Leśniak, 2017). However, mutated cells that break through these protective systems can become proliferative, ultimately leading to cancer. In contrast, neurons rarely acquire proliferative potential. Thus, it is likely that abnormal neurons will continue to reside in the brain contributing to neural activity, throughout the individual's neuronal lifespan.

Age-related genomic instability accumulates in neurons. For example, comparative genomic DNA sequencing of the prefrontal cortex and hippocampal neurons of individuals aged 4 months to 82 years reveals an age-dependent mutation (Lodato et al., 2018). Neurons in the brain with age-related neurodegeneration exhibit more mutations than those in healthy brains (Li et al., 2023). The accumulation of genomic DNA damage in neurons results in various neurodegenerative diseases (Rass et al., 2007). Neurons of Alzheimer's disease patients, for example, exhibit an increase in DSBs accumulation in the early stages, as observed in postmortem brain studies (Suberbielle et al., 2013; Wu et al., 2021; Madabhushi et al., 2015; Reid et al., 2021). Multiple reports have suggested that age-related loss of genomic integrity presumably contributes to Alzheimer's pathogenesis (Weissman et al., 2009; Kruman et al., 2004; Iourov et al., 2009; Herrup et al., 2013).

Mutations in the main component of DNA damage response (DDR) molecules are associated with human chromosomal instability syndromes (McKinnon, 2017; Shiloh, 2003). For example, telangiectatic ataxia is caused by mutations in ATM gene, which is a DDR molecule in DSBs (Shiloh and Rotman, 1996). Mutations in the MRE11, which act in SSBs detection have also been reported to occur in telangiectasia ataxia-like syndrome (Stewart et al., 1999). Similarly, mutations in the XRCC1 gene involved in SSBs repair cause oculomotor palsy, axonal neuropathy, and progressive cerebellar ataxia (Hoch et al., 2017; O'Connor et al., 2018).

## 3 Protection mechanisms of neuronal genome integrity

**Figure 1:**
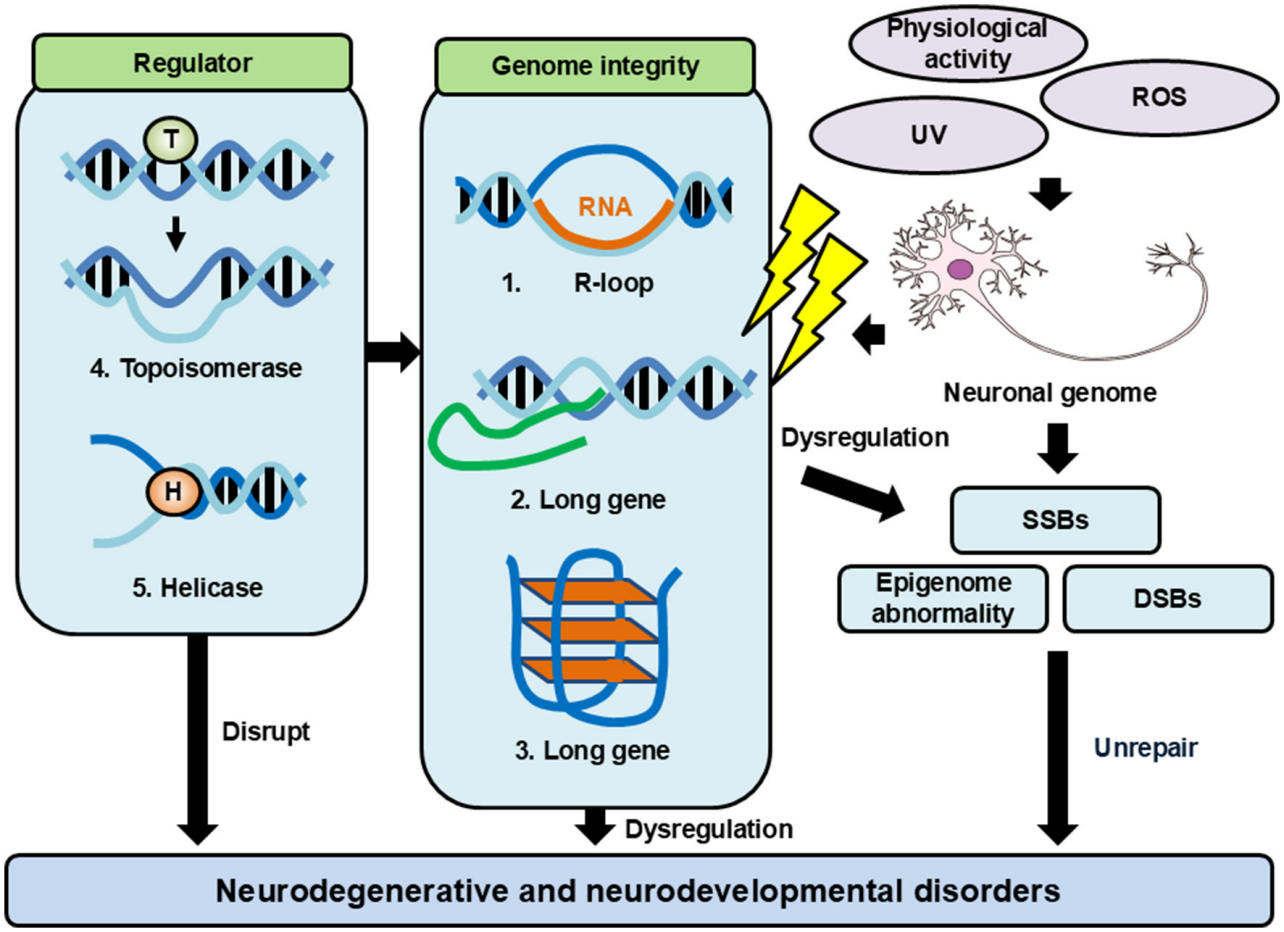
Factors affecting genomic integrity and regulatory mechanisms. Neurons have a lot of events that give damage to genome DNA. These damages cause genomic DNA aberrations and genomic instability. Neurons have many mechanisms to maintain their genomic integrity. When these mechanisms are failed, they lead to diseases such as neurodegenerative and neurodevelopmental disorders.

As described above, functional neurons are frequently damaged by cellular metabolism, neurotransmission, and the regulation of gene expression, which are responsible for the physiological function of a healthy brain. To ensure the long-term maintenance of genomic integrity and neuronal functionality, neurons may rely on specific maintenance mechanisms [57]. Recent evidence from disease associations and experimental studies has shown that DNA structures such as R-loops, G-quadruplexes, and “long genes” influence the neuronal genomic integrity, and these structures are tightly regulated by enzymes such as topoisomerases and helicases (Figure 1).

### 3.1 R-loop

During gene expression, a DNA:RNA hybrid is formed between the template genomic DNA and the nascent RNA transcript, leaving non-template DNA single-stranded. This structure is called R-loop. If R-loop is not properly deleted by Senataxin or RNase H, it causes aberrant of replication forks and lead to DNA damage accumulation. In addition, R-loops affect various biological processes, including transcription, translation, and DNA repair mechanisms. Dysregulation of R-loop is involved in several neurological diseases (Groh and Gromak, 2014; Skourti-Stathaki et al., 2011; Sollier and Cimprich, 2015; Loomis et al., 2014). Abnormal accumulation of R-loop contributes to disorders such as telangiectatic ataxia (Groh and Gromak, 2014; García-Muse and Aguilera, 2019), amyotrophic lateral sclerosis (ALS; Salvi and Mekhail, 2015), ataxia-oculomotor apraxia (Fogel et al., 2014; Becherel et al., 2015), and spinal muscular atrophy (Kannan et al., 2018; Hensel et al., 2020). Recent studies have shown that R-loop contributes to the regulation of NPAS4 expression in response to chronic psychosocial stress or cocaine exposure (Akiki et al., 2024). Thus, R-loop also functions in the immediate response of neural activity, and other physiological functions of R-loop will be elucidated in the future.

The single-stranded DNA within R-loop is vulnerable to SSBs due to its susceptibility to nucleases (Allison and Wang, 2019). Additionally, R-loops create regions of negative and positive supercoiled DNA structures near transcription start sites, forming barriers to transcription elongation (Zardoni et al., 2021), participating in epigenomic regulation (Ginno et al., 2012), and influencing the DNA repair pathway (Keskin et al., 2014). To overcome this barrier, cells transiently cleave and rejoin DNA strands by topoisomerases to relieve torsional stress (Saunders et al., 2006; Le et al., 2019).

In fission yeast (Ohle et al., 2016) and human immortalized cell lines (RPE-hTERT cells; Yasuhara et al., 2018), RAD52 recognizes the R-loop as a landmark for repair, while XPG helicase removes it to activate homologous recombination repair (HRR). However, neurons lack efficient HRR mechanisms for DNA repair. For example, DSBs repair is reduced in sporadic ALS due to abnormal subcellular localization of TDP-43 in motoneurons. TDP-43 is rapidly accumulated at sites of DSBs in neurons and assembles factors that act on DNA repair, particularly non-homologous end-joining (NHEJ; Orii et al., 2006; Mitra et al., 2019). This observation suggests that the molecular machinery is one of the DNA repair mechanisms in R-loop metabolism in neurons.

### 3.2 G-quadruplex

G-quadruplex (G4) is the higher-order structure of nucleic acids which is formed in Hoogsteen hydrogen bonds of guanine (Monsen et al., 2022). These structures have high structural stability and play important functions in the regulation of transcription, replication, DSB site determination, genome stability, and RNA metabolism (Hänsel-Hertsch et al., 2017; Fay et al., 2017). Especially in the neurons, G4 functions to regulate the expression of downstream molecules of genes, such as the promoter of Tyrosine hydroxylase, the rate-limiting enzyme for catecholamine neurotransmitter biosynthesis (Banerjee et al., 2014). In fact, selective disruption of these G4 by mutating promoter DNA sequences affects Tyrosine hydroxylase transcription (Banerjee et al., 2014). Immunohistochemical analysis using adult mouse brains reveals that G4 is widely distributed in neurons throughout the brain regions, including the olfactory bulb, pyramidal cells in the hippocampus, granule cells in the dentate gyrus, and Purkinje cells in the cerebellum (Asamitsu et al., 2020; Comptdaer et al., 2024). Interestingly, G4 distribution in the nucleus of neurons is highly dynamic. G4 immunostaining revealed a lower positive number of G4 in glial cells than in neurons, suggesting that G4 formation is particularly active in neurons (Asamitsu et al., 2020).

Structural analysis indicated that DHX36 helicase, which belongs to the DExD/H box family, resolves the G4 structure (Chen et al., 2018). Genome-wide detection of G4 structure by G4-DNA sequencing, DHX36 unravels the G4 structure and restores the expression of genes suppressed during fear memory in mice exposed to electric shocks paired with sound stimuli, followed by the subsequent fear memory induced upon exposure to sound stimuli alone (Marshall et al., 2024). However, in neuronal progenitor cells, G4 stabilization promotes apoptosis due to DNA damage (Watson et al., 2013). Similarly, in rat neurons, G4 stabilization suppresses Brca1 gene expression, which is essential for DNA repair, and causes the accumulation of DSBs (Moruno-Manchon et al., 2017). Mutations in the ATRX gene stabilize G4 and accumulate DNA damage (Wang et al., 2019). G4 can be detected by gel-shift assay. ATRX helicase binds togenomic DNA and has been suggested to play a role in resolvinggenome-wide G4 and alleviating their adverse effects of G4 (Law et al., 2010).

### 3.3 “Long gene”

Defects in MECP2, a protein that represses gene expression by binding to methylated DNA, result in impaired synaptic function and cause Rett syndrome. Studies using mouse models and brains of Rett syndrome patients have revealed that the MECP2 deficiency increased the expression of “long genes” spanning more than 100 kilobases across the genome, which encode synapse-related genes (Sugino et al., 2014; Gabel et al., 2015). Notably, neurons exhibit significantly higher expression of “long genes” than other cell types (Gabel et al., 2015). “Long genes” are strongly expressed in the frontal lobe and amygdala, which are associated with neurodevelopmental disorders such as autism (Gabel et al., 2015). Also, the expression of “longer genes” is specific to neurons among the cells that compose the brain (Zylka et al., 2015).

“Long genes” have also been implicated in other neurological diseases. TDP-43 and FUS/TLS loci are the genes responsible for amyotrophic lateral sclerosis and transcribe RNA products exceeding 100 kilobases in length (Lagier-Tourenne et al., 2012; Polymenidou et al., 2011). Similarly, CNTNAP2, another “long gene,” has been implicated in autism (Peñagarikano and Geschwind, 2012). Topoisomerase inhibitor topotecan treatment reverses overexpression of “long genes” in Rett syndrome models (Mabb et al., 2014; King et al., 2013).

In Drosophila neurons, aging causes an accumulation of R-loop in the “long gene” and this topological stress is resolved by Top3B (Jauregui-Lozano et al., 2022). “Long genes” make particularly complex DNA and RNA tangles in the regulation of the genome which is a characteristic of neurons. To maintain the genomic integrity of neurons, topoisomerase and various helicase complexes work together regulating “long gene” inducing SSBs and DSBs (Zagnoli-Vieira and Caldecott, 2020).

### 3.4 Topoisomerase

Topoisomerase (Top) works extensively to stabilize the genome and relieve topological stress on the DNA strand. A significant amount of research highlights the Top function in resolving DNA strand breaks during DNA replication to eliminate the super helix structure. Top inhibitors are widely studied for their anticancer effects because they produce DNA breaks that are lethal to proliferative cells (Pommier et al., 2022). Even in non-proliferative neurons, the Top is important for genomic integrity that works to resolve R-loop and G4 structures formed during gene expression. Dysregulation of various Top enzymes is involved in neurodegenerative and neurodevelopmental disorders such as autism, intellectual disorder, schizophrenia, and dementia (Katyal et al., 2014; Neale et al., 2012; Stoll et al., 2013; Tiwari and Wilson, 2019; Fragola et al., 2020; Milano et al., 2024; Crewe and Madabhushi, 2021). For example, Top3B mutations are associated with autism (Stoll et al., 2013; Iossifov et al., 2012), mental disorders (Ahmad et al., 2017a; Stoll et al., 2013), schizophrenia (Xu et al., 2012), and cognitive dysfunction (Kaufman et al., 2016).

Topoisomerase 2β (Top2B) also plays a unique role in neurogenesis. While deletion of Top2B does not affect neuronal production, it disrupts axon outgrowth of ventral horn motor neurons in the spinal cord (Yang et al., 2000). Consistently, Top2B deficient embryonic stem cells show no defects in proliferation or neuronal differentiation (Tiwari et al., 2012). Top2B inhibitor treatment increases the expression of 18% of genes expression in cerebellar granule cells (Tsutsui et al., 2001). In stimulated neurons, Top2B regulates gene expression by inducing DBS into IEGs (Delint-Ramirez et al., 2022). Therefore, regulation of genomic DNA structure by Top is essential for neuronal differentiation and functions.

Topoisomerase 1 (Top1) plays roles during transcription by resolving DNA supercoil, which promotes R-loop formation (Drolet et al., 1995; El Hage et al., 2010). However, Top1 deficiency also increases topological stress and promotes R-loop formation, suggesting that the Top enzyme acts in both R-loop formation and resolution (Promonet et al., 2020). Top3B, which is classified as a Type I Top like Top1, is unique as it can act on both DNA and RNA (Ahmad et al., 2017a,b; Saha et al., 2020). It is known that the functional inhibition of Top3B impairs the R-loop, resulting in a decrease in neuronal function, whereas overexpression of Top3B results in an increase in neuronal function (Skourti-Stathaki and Proudfoot, 2014). Mutant Top3B with reduced enzyme activity causes R-loop accumulation in the genome (Huang et al., 2018). Once engaged with R-loop, Top3B interacts with DDX5 to dissolve the structure (Saha et al., 2022a).

Top enzymes also interact with DNA via cleavage of complexes. For example, Top1 covalently binds to the 3′ phosphate terminus of DNA when DNA is untwisted, and this reaction intermediate (complex with DNA) is called Top1 cleavage complex (Topoisomerase1 cleavage complex; Top1cc), and the complex of Top2 and DNA is called Top2cc (Wojtaszek and Williams, 2024). If unresolved during DNA repair, these complexes can lead to additional DNA damage. Top1cc is removed from DNA by the enzymatic activity of tyrosyl DNA phosphodiesterase 1 (TDP1), and mutations in TDP1 cause spinocerebellar degeneration (El-Khamisy et al., 2005; Takashima et al., 2002). Similarly, mutations in TDP2 which remove Top2cc, are also linked to spinocerebellar degeneration (Gómez-Herreros et al., 2014).

### 3.5 Helicase

Helicase is an enzyme that cuts the hydrogen bonds between the bases of DNA and RNA chains in an ATP-dependent manner and dissociates the nucleic acid strands. In neurons, specific helicases such as DHX36 and ATRX are responsible for the G4 structure in the genome, while DDX5 works to resolve the R-loop (Saha et al., 2022a). Notably, reduced expression of DDX21 in primarily cultured cortical neurons has been shown to accumulate G4 and genomic DNA damage (Lyu et al., 2022). DDX21 also plays a role in eliminating R-loops (Song et al., 2017). Since helicases can unwind both DNA-DNA and DNA-RNA hybrids, they are important factors for maintaining genomic integrity. However, the functional specificity of approximately 100 helicases in the human genome is still largely unknown. Many helicases work in cooperation with Top, suggesting that these complexes form to preserve genomic integrity (Tsukada et al., 2024; Tan et al., 2023; Saha et al., 2022b; Gupta et al., 2022; Yang et al., 2020). Unique and/or specific functions of each helicase should be unraveled in the future.

## 4 Conclusion

The genomic integrity that sustains neuronal function must be maintained for the healthy brain. However, genomic destabilization is an ongoing challenge, arising from daily stress, the physiological activity of neurons, and the accumulation of DNA damage (Zolzaya et al., 2024). Mutations can also occur in the genomes of neurons in normal growth and aging of individuals. In other words, the repair of the neuronal genomic DNA is often incomplete, causing unavoidable genomic damage and possible effects on neuronal function. This ongoing struggle with long-lived neurons during the growth and aging process contributes to the risk of neurodegenerative and psychiatric diseases, where repeated DNA damage and structural genomic changes impair neural function.

Recent studies have suggested that the protective mechanism for neuron-specific genomic integrity may be the action of a large molecular network of topoisomerases, helicases, and factors involved in DNA repair in response to changes in the genomic DNA structure of neurons, including R-loop, G4, and “long gene” regulation. Technological innovations have made it possible to analyze the function of these molecules in a whole genome, and advances in mass spectrometry-based methods have made it possible to identify larger molecular networks comprehensively. By analyzing molecules essential for genomic integrity, we can examine how the molecular networks are responsible for neuronal function throughout the genome, thereby revealing the dynamism of the neuronal genome and linking malfunctions of this network to a variety of neurological diseases.
